# Longitudinal evaluation of quality of life in 288 patients with neurofibromatosis 2

**DOI:** 10.1007/s00415-014-7303-1

**Published:** 2014-03-12

**Authors:** Rosalie E. Ferner, Adam Shaw, D. Gareth Evans, Dympna McAleer, Dorothy Halliday, Allyson Parry, F. Lucy Raymond, Juliette Durie-Gair, C. Oliver Hanemann, Rachel Hornigold, Patrick Axon, John F. Golding

**Affiliations:** 1 National Neurofibromatosis 2 Service, England, UK; 2 Department of Neurology, Guy’s Hospital, Guy’s and St. Thomas’ NHS Foundation Trust, Great Maze Pond, London, SE1 9RT UK; 3 Department of Clinical Neuroscience, Institute of Psychiatry, Kings College London, London, UK; 4 Department of Genetics, Guy’s and St. Thomas’ NHS Foundation Trust, London, UK; 5 Department of Medical Genetics, Central Manchester University Hospitals Foundation Trust, Manchester, UK; 6 Department of Clinical Genetics, Oxford Radcliffe Hospitals NHS Trust, Oxford, UK; 7 Department of Neurology, Oxford Radcliffe Hospitals NHS Trust, Oxford, UK; 8 Department of Medical Genetics, Addenbrooke’s Hospital, University of Cambridge, Cambridge University Hospitals NHS Foundation Trust, Cambridge, UK; 9 Department of Skull Base Surgery, Addenbrooke’s Hospital, Cambridge University Hospitals NHS Foundation Trust, Cambridge, UK; 10 Centre for Biomedical Research, Peninsula Schools of Medicine and Dentistry, Plymouth University, Plymouth, UK; 11 Department of Psychology, University of Westminster, London, UK

**Keywords:** Neurofibromatosis 2, NF2, NFTI-QOL, Vestibular schwannoma

## Abstract

Advances in molecular biology have resulted in novel therapy for neurofibromatosis 2-related (NF2) tumours, highlighting the need for robust outcome measures. The disease-focused NF2 impact on quality of life (NFTI-QOL) patient questionnaire was assessed as an outcome measure for treatment in a multi-centre study. NFTI-QOL was related to clinician-rated severity (ClinSev) and genetic severity (GenSev) over repeated visits. Data were evaluated for 288 NF2 patients (*n* = 464 visits) attending the English national NF2 clinics from 2010 to 2012. The male-to-female ratio was equal and the mean age was 42.2 (SD 17.8) years. The analysis included NFTI-QOL eight-item score, ClinSev graded as mild, moderate, or severe, and GenSev as a rank order of the number of NF2 mutations (graded as mild, moderate, severe). The mean (SD) 8.7 (5.4) score for NFTI-QOL for either a first visit or all visits 9.2 (5.4) was similar to the published norm of 9.4 (5.5), with no significant relationships with age or gender. NFTI-QOL internal reliability was good, with a Cronbach’s alpha score of 0.85 and test re-test reliability *r* = 0.84. NFTI related to ClinSev (*r* = 0.41, *p* < 0.001; *r* = 0.46 for all visits), but weakly to GenSev (*r* = 0.16, *p* < 0.05; *r* = 0.15 for all visits). ClinSev related to GenSev (*r* = 0.41, *p* < 0.001; *r* = 0.42 for all visits). NFTI-QOL showed a good reliability and ability to detect significant longitudinal changes in the QOL of individuals. The moderate relationships of NFTI-QOL with clinician- and genetic-rated severity suggest that NFTI-QOL taps into NF2 patient experiences that are not encompassed by ClinSev rating or genotype.

## Introduction

Neurofibromatosis 2 (NF2) is an inherited tumour suppressor disease with a prevalence of 1 in 60,000 and a birth incidence of 1 in 25–33,000 individuals [1, 2]. Bilateral vestibular schwannomas (VS) are emblematic of NF2, but schwannomas may form on other cranial, spinal, and peripheral nerves. Central nervous system meningiomas and ependymomas, peripheral neuropathy, amyotrophy, retinal hamartomas, and subcapsular lens opacities are part of the NF2 disease spectrum [1, 3]. Typically the presenting symptoms in adults are hearing loss and balance disturbance, and these symptoms reflect the major causes of morbidity and impact on quality of life (QOL) in people with NF2 [1, 3, 4]. Impaired vision and facial weakness may compound the problems in individuals who already have to contend with deafness. The *NF2* gene was identified on chromosome 22q11.2 and somatic mosaicism is present in about one-third of de novo patients [1, 2, 5]. Germline truncating mutations are associated with more severe disease than large deletions and missense mutations; individuals with germline truncating mutations are diagnosed at a younger age and usually have an earlier onset of symptomatic tumours [6, 7]. Recent developments in neurosurgery, radio-surgery, auditory rehabilitation, and molecular biology have increased the treatment options for individuals with NF2 [8]. Bevazicumab acts as a vascular endothelial growth factor inhibitor that reduces vestibular schwannoma growth and shrinks tumours in some patients; other novel drugs are being investigated in clinical trials, including lapatinib, a tyrosine kinase inhibitor, RAD0001 [9–11]. However, randomised controlled therapeutic trials, the gold standard of efficacy, are difficult to undertake because of the rarity of NF2. Assessment of genotype, in combination with clinician- and patient-rated severity is useful to determine therapeutic outcomes. Meticulous clinical evaluation includes neurological and visual examination, timed gait assessment, neurophysiology, speech and pure tone audiometry, and serial, standardised measurement of tumours on 1-mm magnetic resonance imaging [1, 8]. Few studies have addressed QOL in people with NF2. Neary et al. [12] reported that the predominant problems were impaired balance and difficulty with social communication when they used a closed-set questionnaire and the generic Short Form-36 (SF-36) questionnaire to evaluate QOL in NF2 patients [12, 13]. Patel undertook semi-structured interviews in six patients and revealed that NF2 had a negative impact on daily activities including employment, and was associated with social isolation arising from communication difficulties [4]. Family played a central role in providing physical, psychological, and emotional support. We developed the NF2 Impact on Quality of Life (NFTI-QOL) questionnaire, a reliable, validated disease-focused assessment for NF2 patients, for health-care providers to use as a clinical assessment tool and outcome measure (Table 1) [14]. The questionnaire is completed in a few minutes and comprises eight questions, with a maximum score of 24 reflecting the greatest impact on QOL. There is a free-response section at the end if individuals wish to add expand an answer and add new information. NFTI-QOL covers the domains of balance and dizziness, hearing, facial weakness, sight, mobility and walking, role and outlook on life, pain, anxiety, and depression [14] (Table 1).

**Table 1:**
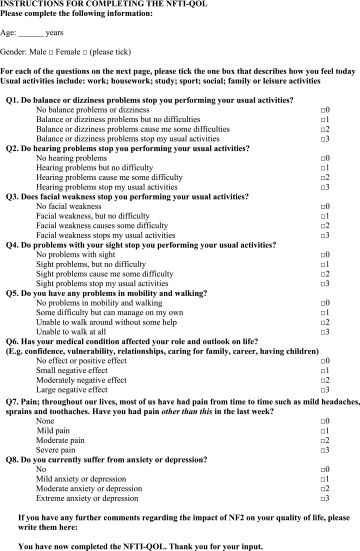
NFTI-QOL English version for the UK (neurofibromatosis 2 impact on quality of life) (reproduced with permission from Hornigold et al. [14])

## Aims

The aim of this study was to evaluate the NFTI-QOL as an assessment tool and potential outcome measure for therapeutic intervention in multi-centre national NF2 clinical services. Patient-reported experience using the NFTI-QOL was related to clinician-rated and genetic severity (GenSev) scores. Changes over repeated clinic visits were evaluated to detect stable disease and changes after intervention.

## Methods

### Ethical approval

The study was approved as a clinical evaluation, with study number 3711, by the Clinical Audit Group committee at Guy’s and St Thomas’ National Health Service (NHS) Foundation Trust, London.

### Participating centres, inclusion and exclusion criteria

NIFTI-QOL data were collected for all patient visits to the national NF2 services at Guy’s and St. Thomas’ NHS Foundation Trust, Oxford University Hospitals NHS Trust, Addenbrooke’s Hospital, Cambridge University NHS Foundation Trust, Central Manchester University Hospitals Foundation Trust, and satellite NF2 services from April 2010 to October 2012. Individuals who did not fulfil the diagnostic criteria for generalised or mosaic NF2, or who were aged <16 years were excluded from the study. Age and gender were recorded for all patients.

### Clinician-rated severity

Clinician-rated severity was assessed as severe, moderate, or mild and was determined during the clinical visit. Severe disease was classified as symptomatic presentation at age <20 years with at least two symptomatic or large tumours >1.5 cm, in addition to VS and including tumours removed previously. Individuals who were diagnosed with a central nervous system tumour before the age of 12 years and had at least one other symptomatic tumour were also rated as severe. Patients older than 30 years at presentation with no more than two symptomatic or large tumours >1.5 cm including VS and tumours excised previously were assessed as mild. Moderate disease was classified as not meeting mild or severe criteria [1, 3].

### Genetic-rated severity

Genetic severity was rated as severe, moderate, or mild. Truncating mutations in exons 1–13 in all cells were rated as severe. Moderate disease included (1) deletion not involving the promoter region or exon 1, (2) splice site mutations in exons 1–8, and (3) mosaicism of truncating mutations in exons 1–13 in blood. Mild disease was classified as (1) a missense mutation or an in-frame deletion, (2) a large deletion involving the promoter region or exon 1, (3) a splice-site mutation in exons 9–15, (4) mosaicism (excluding moderate criteria), and (5) no mutation identified on blood analysis [1, 6, 7, 15, 17–19].

### Clinical assessment and NFTI-QOL

At each clinic visit, patients underwent clinical assessment and completed the NFTI-QOL, an eight-item questionnaire (and a free-response section) with a maximum score of 3 per item (four-point scale, range 0–3, with three as the most impaired) and total score of 24 [14]. Patients who were unable to complete the questionnaire due to visual or motor difficulties were assisted by a specialist nurse.

### Statistics

The NFTI-QOL, clinical, and genetic data were analysed with SPSS using correlation, linear regression, and analysis of variance (ANOVA) tools as required.

## Results

### Patient and visit numbers

Data were evaluated for 288 NF2 patients attending the national NF2 clinics and satellite centres in England (London, Oxford, Cambridge, Manchester) from 2010 to 2012. In total, there were 464 patient visits, including 288 (62.1 %) attending one visit, 117 (25 %) attending two visits, 41 (8.9 %) with three visits, 14 (3 %) with four visits, two patients (0.4 %) having five visits, and two (0.4 %) attending six visits.

### Gender, age, clinical- and genetic-rated severity

The studied group was comprised of 143 males and 145 females; the mean age was 42.2 years (SD 17.8) and the age range was 16–87 years. There were no significant differences in age or gender of the patients attending the four different NF2 centres. Clinical- and genetic-rated severity for 288 individuals with NF2 is shown in Table 2. Thirty patients elected not to have genetic testing. There were no significant differences in genetic-rated severity between the four centres, but mean clinical severity (ClinSev) was milder in the Manchester centre (1.5) than in London (2.0), Cambridge (2.0), or Oxford (1.9) (Kruskal Wallace test, *df* 3, *χ* ^2^ 24.8, *p* < 0.01).

**Table 2:** Clinical- and genetic-rated severity in 288 NF2 patients

Severity	Clinical severity, *n* (%)	Genetic severity, *n* (%)
Mild	124 (43.1)	168 (58.4)
Moderate	93 (32.3)	49 (17)
Severe	70 (24.3)	41 (14.2)
Not tested	0	30 (10.4)
Not classified	1 (0.3)	0 (0)

### NFTI-QOL

**Fig. 1:**
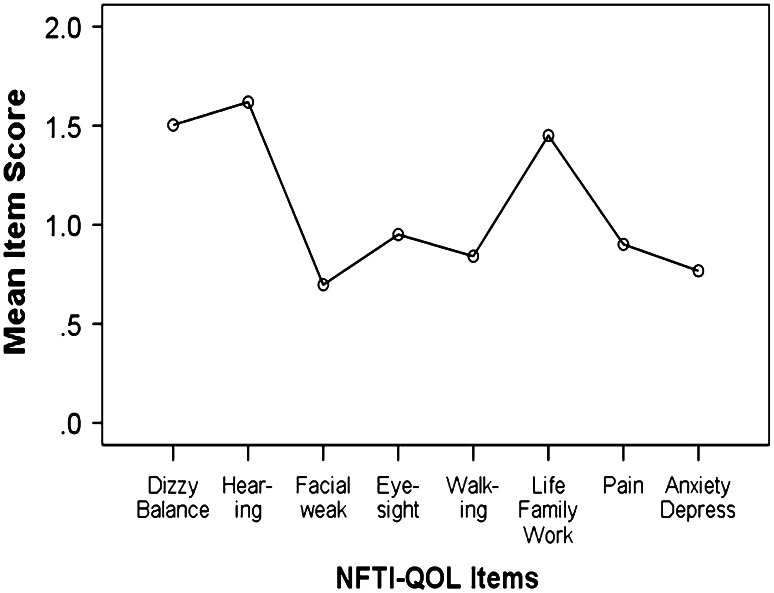
Mean item scores for NFTI-QOL in 288 patients on first visit to London, Oxford, Cambridge, or Manchester NF2 centre

**Fig. 2:**
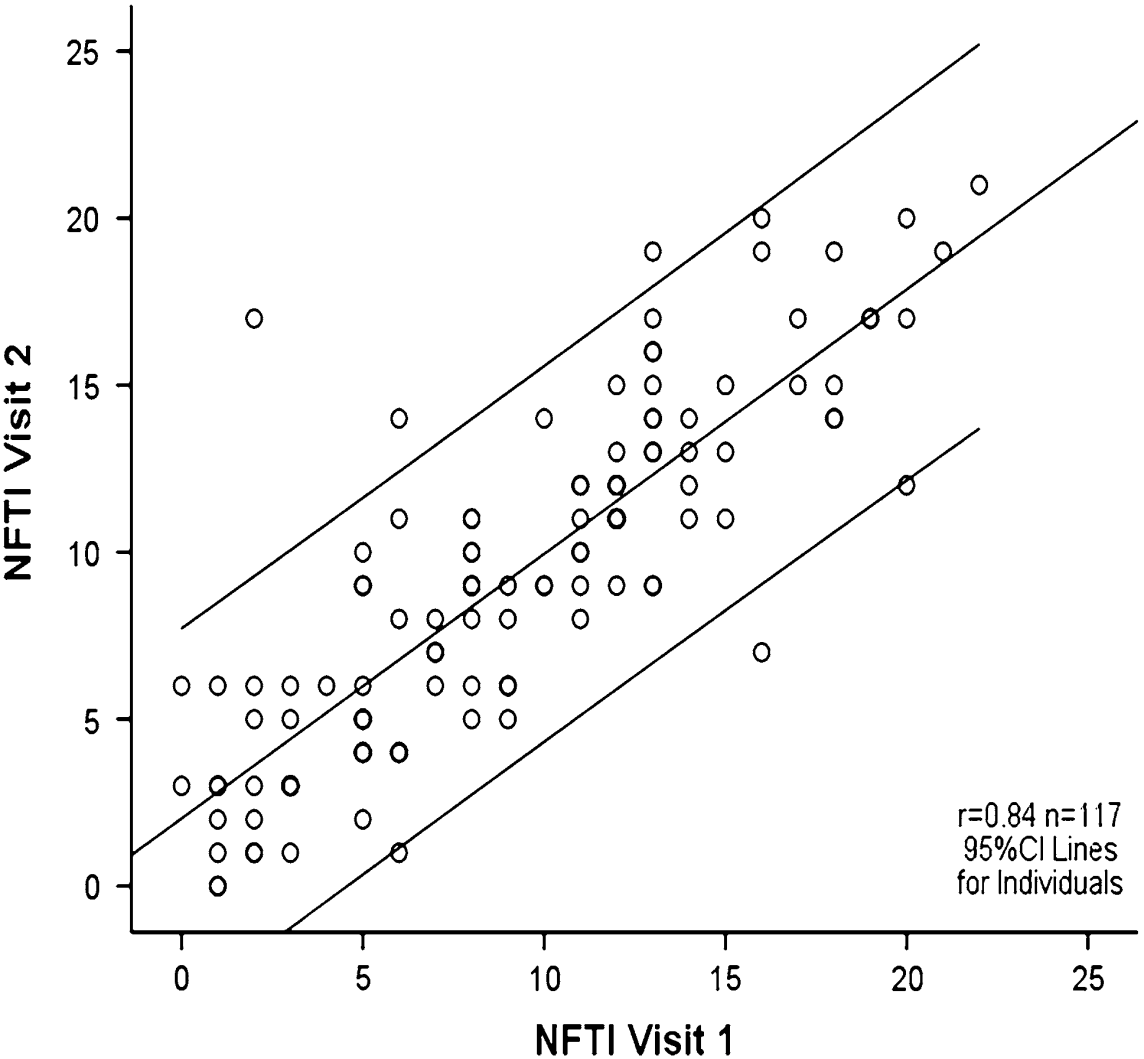
NFTI-QOL scores are shown for NF2 individuals who attended both visit 1 and visit 2 (*n* = 117). The *thick line* is the fitted regression line and the *thin lines* on either side denote a 95 % CI for individuals. The *darker circles* indicate superimposed observations for multiple NF2 individuals

The mean NFTI-QOL score for 288 patients for the first clinic visit was 8.7 (SD 5.4), for the 117 patients who attended on a second visit it was 9.3 (SD 5.2), and for all visits the mean NFTI-QOL was 9.2 (SD 5.4). This was similar to the published norm of 9.4 (5.5), with no significant relationships with age or gender. Analysis of variance (ANOVA) revealed no differences in NFTI-QOL scores for the four NF2 sites. Hearing loss, dizziness and balance, and impact of NF2 on role and outlook on life were the items that showed the highest severity ratings for all centres (Fig. 1). The evaluation of patient-rated QOL (London) from visit 1 to visit 2 showed that the majority of patients had stable disease. However, three patients were significantly worse and three showed significant improvement, as demonstrated by the 95 % confidence intervals (CIs) for individuals with NF2 (Fig. 2). Examination of longitudinal changes indicated that any NFTI-QOL score change up or down of greater than five points would be statistically significant for an individual (Fig. 2). The internal reliability of NFTI-QOL was found to be very good, with a Cronbach’s alpha score of 0.85 and re-test reliability *r* = 0.84. The NFTI-QOL was related to ClinSev (*r* = 0.41, *p* < 0.001; *r* = 0.46 for all visits), but was only related weakly to GenSev (*r* = 0.16, *p* < 0.05; *r* = 0.15 for all visits). Clinical severity in turn related to GenSev (*r* = 0.41, *p* < 0.001; *r* = 0.42 for all visits).

### NFTI-QOL free responses

There were infrequent free responses, and where present, they tended to amplify one of the eight NFTI-QOL domains, rather than report new symptoms or problems. One individual rated all domains as 0 on the first clinic visit, despite complaining of worsening hearing, balance, and role and outlook on life in the clinic. On the second visit, staff found that the patient had not learnt to read at school and that the total NFTI-QOL score was actually 6 when appropriate assistance was given for completion of the questionnaire.

## Discussion

In this 2-year longitudinal study, we assessed clinical-, genetic-, and patient-rated disease severity in 288 patients in four NF2 centres in England. We evaluated the mean total and individual item NFTI-QOL scores for the four NF2 centres. This study did not reveal any significant difference in the mean NFTI-QOL or individual item scores. NFTI-QOL showed good internal reliability, good test–retest reliability and demonstrated stability over time. Moreover, the mean NFTI-QOL and individual item scores remained stable for multiple visits, with no particular trend for increase or decrease in items over time. Obviously, there were wide variations in NFTI-QOL scores between individuals, reflecting different perceptions and coping mechanisms in response to chronic disease. Within the context of the overall high reliability and stability of the NFTI-QOL, an increase or reduction in scores was evident in some individuals over time. The total NFTI-QOL score is the optimum measure for estimating an individual’s overall QOL and changes that occur over time. The clinician may find it useful to evaluate the individual item scores to look for changes in a particular domain, as this reflects the relative importance of that domain to the individual. Scores vary with individual circumstances and ability of that individual to cope with the impact of the disease on that particular domain. For example, a 32-year-old male had deteriorating total NFTI-QOL scores (from 13 to 16) until a cochlear implant was inserted and specialist neuro-rehabilitation was undertaken for impaired mobility. The total NFTI-QOL score reduced to 9 and individual item scores improved for hearing, balance, mobility, and role and outlook on life. This indicates the potential for NFTI-QOL to detect changes in disease symptoms or response to treatment. It also underlines the observation that improvement in one domain, for instance hearing, may generalise to improvements in other domains. There was a change of more than five points in the total NFTI-QOL score in five other patients, including four males and one female, aged 19–62 years (median age 38 years). The total NFTI-QOL scores decreased in three individuals due to improved hearing and balance and neurological symptoms. The total NFTI-QOL scores increased in two patients with worsening hearing, balance, and psychological issues that had a deleterious impact on their role and outlook in life. A five-point change or greater was statistically significant for an individual (95 % CI for individuals). However, this is a statistical cutoff and smaller changes in scores could well have clinical significance for an individual (Fig. 2). Although NFTI-QOL correlates with ClinSev, it appears to tap into more about the impact of NF2 on the individual, rather than simply being a reflection of clinical and GenSev. Overall, ClinSev was rated as milder for patients attending the Manchester centre compared with London, Cambridge, and Oxford, and this might simply reflect the disease heterogeneity. There was a weak correlation between NFTI-QOL and GenSev. This could have arisen because GenSev looks at the disease from a long-term perspective, whereas the patient-rated NFTI-QOL mirrors current disease status. The weaker correlation between NFTI-QOL and GenSev might have arisen because some individuals with a mutation predicting severe NF2 were assessed early in the course of their disease and had not yet developed many symptoms or deficits. Furthermore, individuals with genetic mutations associated with mild disease, may still have significant clinical symptoms and neurological deficit, albeit with a lower tumour burden and older age at presentation. One might expect that anxiety and depression would be a major feature of NF2, and this is the case for some. However, the mean item score for anxiety and depression for all visits was low, and this likely reflects the high levels of psychological, psychiatric, and educational support available to people attending the national NF2 centres. Most patients did not have visual problems and the mean NFTI-QOL score for sight was low, but it was reported as a major problem for a minority of individuals particularly when it compounded hearing loss. Disease specific QOL questionnaires are an important tool for evaluating patient focused outcomes of intervention. If a new agent is capable of shrinking a tumour but the patient does not experience an improvement in symptoms or QOL, careful thought should be put into continuing administration of the drug, unless it reduces mortality rates. One of our patients had a significant reduction in size of a VS following 3 months of treatment with bevacizumab; nonetheless, the total NFTI-QOL score remained 22, as the patient had not noted improvement in any of the NFTI-QOL domains. NFTI-QOL is validated for people with NF2 16 years and older, but potentially could be extended for use in 12-year-olds. The cohort of younger children with NF2 is small, and current clinical trials are focused on teenagers and adults. Further work would be required to develop a disease-specific NF2 QOL questionnaire in this younger age group. This is because visual impairment, neurological deficit from amyotrophy, central nervous system ependymomas, and meningiomas may be more prominent findings than hearing, loss of balance, and dizziness from VS [1, 3].

## Conclusions

NFTI-QOL is a disease-specific questionnaire that is quick and easy to administer. It shows good reliability and has the ability to detect significant changes over time in QOL of individual patients. We have demonstrated previously that NFTI-QOL correlates strongly and significantly with all domains of the SF-36 and with the EuroQOL, generic questionnaires that do not focus on NF2-specific problems [13, 16]. The moderate relationship between NFTI-QOL and ClinSev and genetic-rated severity was consistent with the notion that NFTI-QOL draws on other dimensions of NF2 patient experiences that are not covered by either of these latter measures.
